# The accumulation of technical errors exponentially increases the risk of screw cut-out in femoral intramedullary nailing

**DOI:** 10.1007/s00402-026-06263-x

**Published:** 2026-03-09

**Authors:** Damien Brochard, Maëlys Thepaut, Thomas Daoulas, Arthur Poiri, Hoel Letissier, Rémi Di Francia

**Affiliations:** 1 Hospital of the Pays de Morlaix, Morlaix, France; 2 Centre Hospitalier Régional Universitaire de Brest, Brest, France

**Keywords:** Trochanteric femur, tip-apex distance, fracture, osteosynthesis

## Abstract

**Purpose:**

Proximal femur fractures primarily affect the elderly, with significant morbidity, mortality, and socioeconomic impact. The main complication of short trochanteric intramedullary nailing is the cut-out of the cervicocapital screw through the femoral head. The objective of this study was to analyze the influence of technical errors in short trochanteric intramedullary nailing for the treatment of trochanteric femur fractures on the mechanical failure of osteosynthesis.

**Methods:**

A total of 540 patients who underwent surgery for a trochanteric femur fracture using short trochanteric intramedullary nailing were included in a single-center, retrospective study conducted between February 2012 and July 2018.

**Results:**

Thirty patients (5.6%) experienced mechanical failure of the osteosynthesis at the 3-month follow-up. An anterior position of the cervicocapital screw, accumulation of technical errors, a tip-apex distance > 25 mm, and an intra-focal entry point were significantly associated with cervicocapital screw cut-out.

**Conclusion:**

The mechanical failure rate is 5.6%. Short trochanteric intramedullary nailing requires precise execution to reduce the risk of cervicocapital screw cut-out, which is a source of osteosynthesis failure.

## Introduction

Trochanteric fractures are very common, with a prevalence of 75% in people over 65 [1]. This type of injury can be life-threatening and functionally crippling for the elderly. The mortality rate is around 7% at 1 month, 12% to 23% at 6 months, 26% to 30% at one year, and up to 50% in the case of major comorbidities [2, 3]. These fractures therefore represent a major public health issue. Intramedullary osteosynthesis using short intramedullary nail (SIN) is one of the most widely used techniques for treating trochanteric fractures [4]. Although it is considered a common procedure in trauma surgery, the SIN can be performed with a number of technical errors, potentially leading to serious complications such as cut-out of the cervicocapital screw (CCS), defined by a collapse of the cervico-diaphyseal angle into varus, resulting in screw extrusion from the femoral head [5]. The literature shows that the rate of these complications can reach up to 20% of patients [6–9] and can lead to poor functional outcomes [10–12]. Few studies have focused on these errors and their correlation with cervicocapital screw cut-out. Studies by Bojan and Morvan highlighted risk factors related to the positioning of the CCS and the tip-apex distance, but they did not address the cumulative effect of technical errors [5, 13]

The aim of this study was to analyze the influence of the type and number of technical errors during SIN osteosynthesis on the mechanical failure of osteosynthesis in the treatment of trochanteric femur fractures. The hypothesis of this study was that an accumulation of technical errors during SIN osteosynthesis increases the risk of CCS cut-out.

## Patients and methods

### Patients

####  Study design

This was a retrospective, descriptive, single-center, multi-operator study operated on between February 2012 and July 2018.

#### Population

All patients over 18 years of age who presented with a trochanteric fracture treated surgically by SIN during the study period were included.

Patients lost to follow-up and those with a follow-up of less than 3 months were excluded.

##### Informed consent

was obtained orally for all patients.

###  Methods

####  Data collection 

**Fig. 1:**
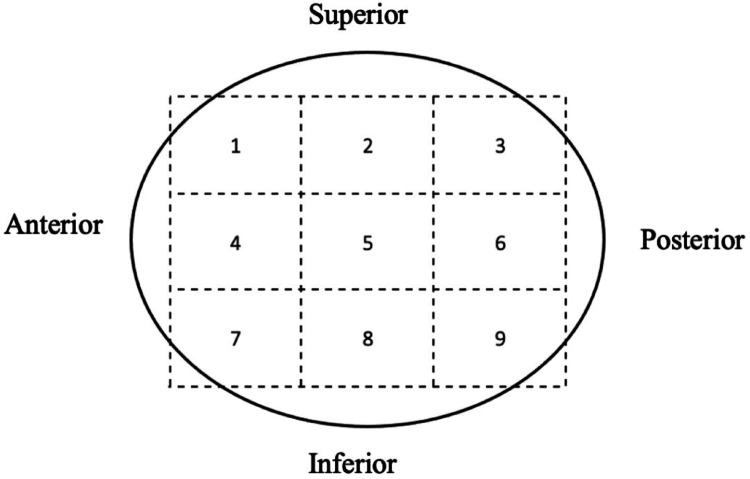
Distribution of zones according to Cleveland

**Fig. 2:**
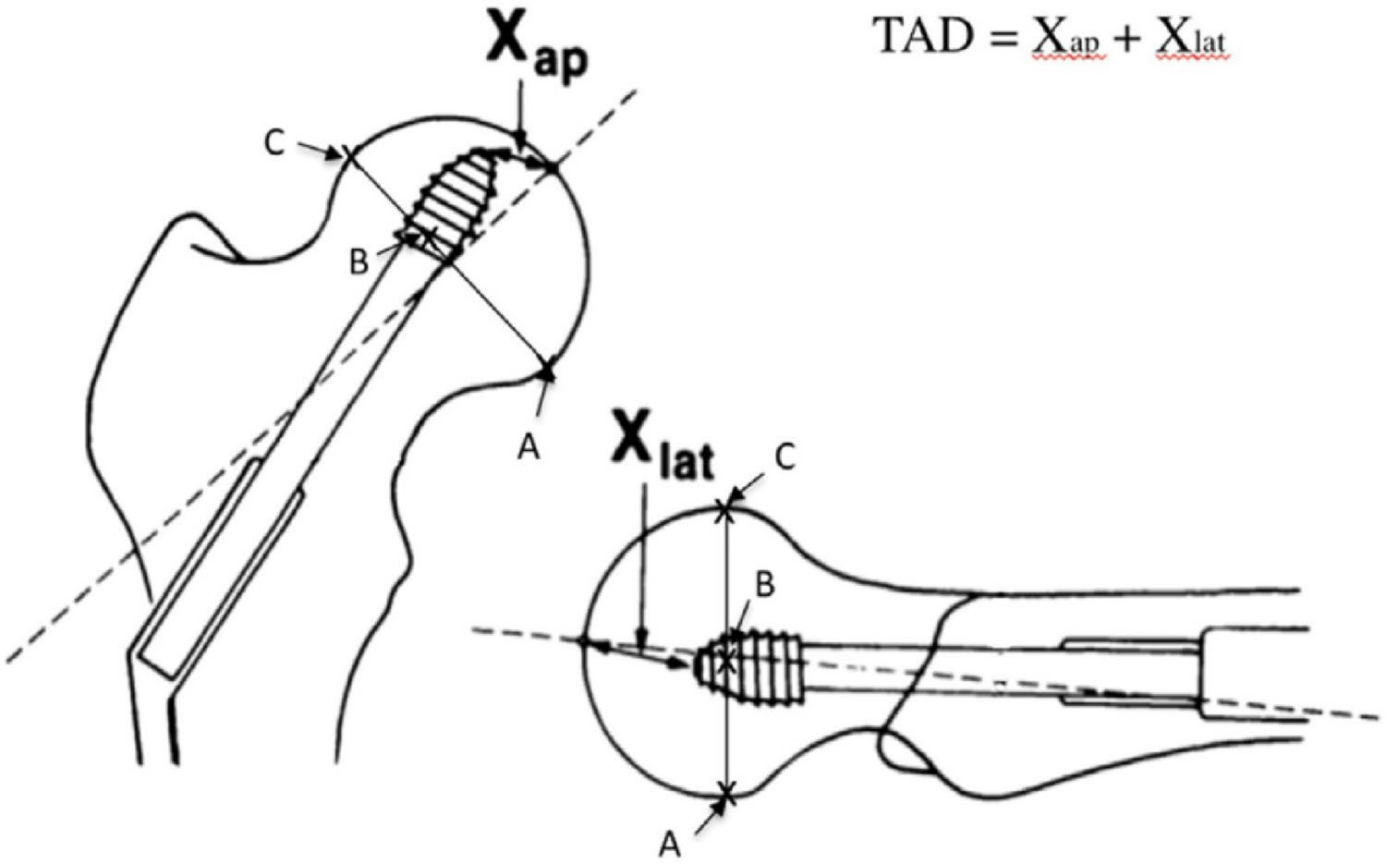
The Tip-Apex Distance (TAD) is the sum of the distances, in millimeters, from the tip of the cervicocapital screw to the femoral head apex on the anteroposterior (Xap) and lateral (Xlat) radiographs

Data were collected with a minimum 3-month follow-up. Age, sex, side of fracture, AO/OTA fracture type, trochanteric nail type and angulation were recorded. Radiographic criteria were reduction quality: “good”, “acceptable” or “poor”, according to Baumgaertner’s method [14], the positioning of the cervicocapital screw (CCS) in the femoral head according to Cleveland zones in the immediate postoperative period (within 48 h after surgery) [15] (Fig. 1), errors in the entry point of the intramedullary nail, errors in the tip-apex distance (Fig. 2), errors in the CCS-cartilage distance, and errors in the distal locking screw.

####  Primary endpoint

The primary endpoint was the occurrence of mechanical failure of osteosynthesis before the third postoperative month, defined by cervicocapital screw cut-out. Two groups were formed based on the presence or absence of cervicocapital screw cut-out. The “cut-out” group (CO) and the “no cut-out” group (NCO) were compared.

#### Secondary endpoints

The secondary endpoints were the number of errors and their distribution according to the entry point, the CCS, the distal screw, as well as the association of errors with screw cut-out.

### Statistical analysis 

For each variable, means and standard deviations were calculated after verifying normality using a Chi-squared test. Means were compared using t-tests, and proportions were compared using Chi-squared tests with a significance level of α = 0.05. Relative risks were calculated both univariately and multivariately. Risk factors with a relative risk showing statistical significance (*p* < 0.05) in the univariate analysis were retained for the multivariate analysis: a multiple regression was performed (retrospective method).

## Results 

1. Patients

**Fig. 3:**
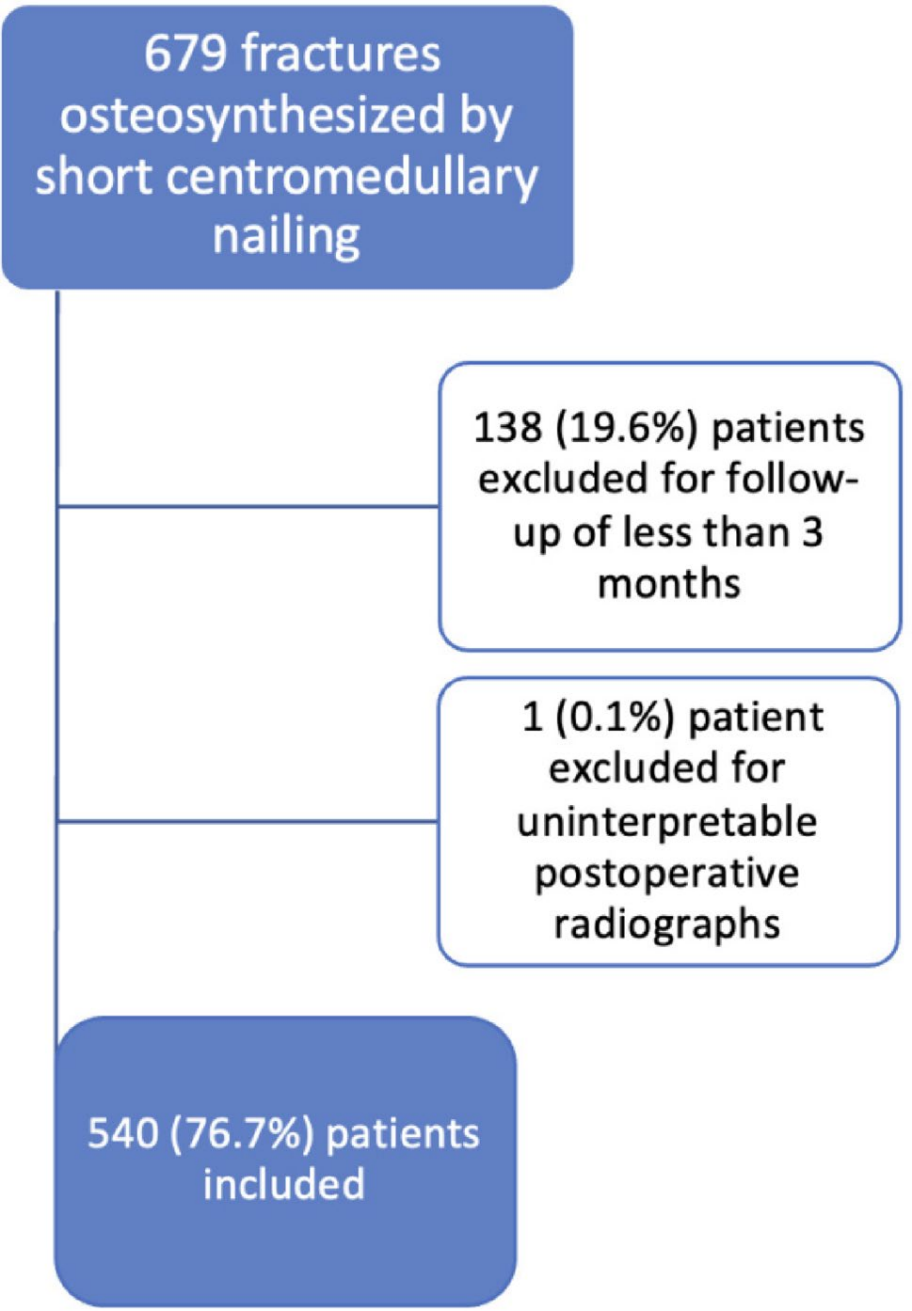
Flowchart

540 out of 679 eligible patients were included and analyzed after the exclusion of 139 patients lost to follow-up with less than 3 months of follow-up (Fig. 3). Group B (*n* = 30) and group AB (*n* = 510) were comparable in terms of fracture type distribution (31A1: *p* = 0.07; 31A2: *p* = 0.06; 31A3: *p* = 0.53; 31B3: *p* = 0.21) and sex (*p* = 0.42). Patients in group B were significantly older (*p* = 0.05).

2. Primary endpoint

Among the 540 included patients, 30 (5.6%) exhibited a cut-out at 3-month follow-up.

3. Univariate analysis (Table 1)

Among the 540 included patients, 30 (5.6%) exhibited a cut-out at 3-month follow-up.

**Table 1:** Univariate Analysis of Predictive Factors for Sweep

Mean age (years) (Min-Max ; standard deviation)	No Cut out group (NCO) *N* = 510	Cut out group (CO) *N* = 30	*p*	RELATIVE RISK
	80,57 (15–100 ; 13,48)	85,47 (68–94 ; 6,38)	*P* = 0.0487	Age > 80 RR = 1.1938 *P* = 0.0410
AO CLASSIFICATION				
31A1	200 (39%)	7 (23%)	*P* = 0,0798	
31A2	221 (43%)	18 (60%)	*P* = 0.0685	
31A3	64 (13%)	5 (17%)	*P* = 0,5300	
31B3	25 (4.9%)	0 (0%)	*P* = 0,2148	
ANGULATION (degrEeS)				
120	8 (1.6%)	0 (0%)	*P* = 0,4855	
125	454 (89%)	30 (100%)	*P* = 0,0552	
130	47 (9.2%)	0 (0%)	*P* = 0,0824	
135	1 (0.2%)	0 (0%)	*P* = 0,8065	
REDUCTION				
NUL	5 (0.98%)	1 (3.3%)	*P* = 0,2387	
ACCEPTABLE	88 (17%)	20 (67%)	*P* < 0,0001	RR = 3.8636 *P* < 0.0001
GOOD	417 (82%)	9 (30%)	*P* < 0,0001	RR = 0.3669 *P* = 0.0003
CLEVELAND				
1 anterosuperior	4 (0.78%)	10 (33%)	*P* < 0.0001 (Chi2)	RR = 42.5 *P* < 0.0001
2 SUPERIoR	25 (4.9%)	1 (3.3%)	*P* = 0,6909	
3 POSTEROSUPERioR	0 (0%)	1 (3.3%)	*P* < 0,0001	RR = 49.4516 *P* = 0.0162
4 ANTERIoR	20 (3.9%)	7 (23%)	*P* < 0,0001	RR = 5.9500 *P* < 0.0001
5 CENTRED	304 (60%)	4 (13%)	*P* < 0,0001	RR = 0.2237 *P* = 0.0013
6 POSTERIoR	65 (13%)	5 (17%)	*P* = 0,5300	
7 ANTEROINFERIoR	2 (0.39%)	0 (0%)	*P* = 0,7321	
8 INFERIoR	64 (13%)	0 (0%)	*P* = 0,0352	RR = 0.1278 *P* = 0.1438
9 POSTEROINFERIoR	27 (5.3%)	2 (6.7%)	*P* = 0,7414	
AT LEAST ONE ERROR NAIL	292 (57%)	30 (100%)	*P* < 0,0001	RR = 1.7466 *P* < 0.0001
ERROR NUMBER				
MEAN	0,961 (0–6 ; 1,07)	3,00 (1– 5 ; 1,17)	*P* < 0,0001	
0	218 (43%)	0 (0%)	*P* < 0,0001	RR = 0.03772 *P* = 0.0195
1	160 (31%)	2 (6.7%)	*P* = 0,0047	RR = 0,2125 *P* = 0.0240
2	82 (16%)	10 (33%)	*P* = 0,0160	RR = 2,0732 *P* = 0.0086
3	36 (7.1%)	8 (27%)	*P* = 0,0001	RR = 3.7778 *P* = 0.0001
4	13 (2.5%)	6 (20%)	*P* < 0,0001	RR = 7.8462 *P* < 0.0001
5	0 (0%)	4 (13%)	*P* < 0,0001	RR = 148.3548 *P* = 0.0007
6	1 (0.2%)	0 (0%)	*P* = 0,8065	
AT LEAST ONE ERROR ENTRY POINT	130 (25%)	19 (63%)	*P* < 0,0001	RR = 2,4846 *P* < 0,0001
TOO ANTERIoR	4 (0,74%)	0	*P* = 1	
TOO latéral	15 (2,9%)	6 (20%)	*P* < 0,0001 (Chi2)	RR = 6.8 *P* < 0.0001
TOO MEDIAL	19 (3.7%)	1 (3.3%)	*P* = 0,9122	
TOO postérior	76 (15%)	9 (30%)	*P* = 0,0289 (Chi2)	RR = 2,0132 *P* = 0.0190
Intrafocal	37 (7%)	11 (37%)	*P* < 0.0001 (Chi2)	RR = 5.05 *P* < 0.0001
NUMBER OF ERRORS ENTRY POINT				
0	380 (75%)	11 (37%)	*P* < 0,0001	RR = 0,4
1	109 (21%)	11 (37%)	*P* = 0,039	RR = 1,71
2	21 (4.1%)	8 (27%)	*P* < 0,0001	RR = 6,4
AT LEAST ONE ERROR CCS	292 (57%)	30 (100%)	*P* < 0.001	RR = 1.74 *P* < 0.0001
ERROR NUMBER CCS				
0	269 (53%)	0 (0%)	*P* < 0,0001	RR = 0,03058 *P* = 0.0130
1	169 (33%)	7 (23%)	*P* = 0,2560	
2	63 (12%)	14 (47%)	*P* < 0,0001	RR = 3.7778 *P* < 0.0001
3	9 (1.8%)	8 (27%)	*P* < 0,0001	RR = 15,1111 *P* < 0.0001
4	0 (0%)	1 (3.3%)	*P* < 0,0001	RR = 49,4516 *P* = 0.0162
supeRIor	29 (5,6%)	12 (40%)	*P* < 0,0001	RR 7,1034 *P* < 0,0001
TOO ANTERIor	26 (5.1%)	17 (57%)	*P* < 0,0001	RR = 3.1413 *P* < 0.0001
TOO POSTERIor	92 (18%)	8 (27%)	*P* = 0,2177	
TAD (mm)	19,92 (0–50 ; 5.90)	24.87 (6–45 ; 8.09)	*P* < 0,0001	
TOO SHORT TAD > 25 mm	86 (17%)	14 (47%)	*P* < 0,0001 (Chi2)	RR = 2.7674 *P* < 0.0001
TOO long (TAD <5 mm)	36 (7,1%)	7 (23%)	*P* = 0,0018 (Chi2)	RR = 3,3056 *P* = 0,0012
NUMBER OF ERRORS Distal screw				
0	493 (97%)	30 (100%)	*P* = 0,3363	
1	16 (3.1%)	0 (0%)	*P* = 0,3281	
2	1 (0.2%)	0 (0%)	*P* = 0,8065	

TAD : tip apex distance

CCS: cervicocapital screw

RR : relative risk

CO: cut-out

NCO : no cut-out

**Fig. 4:**
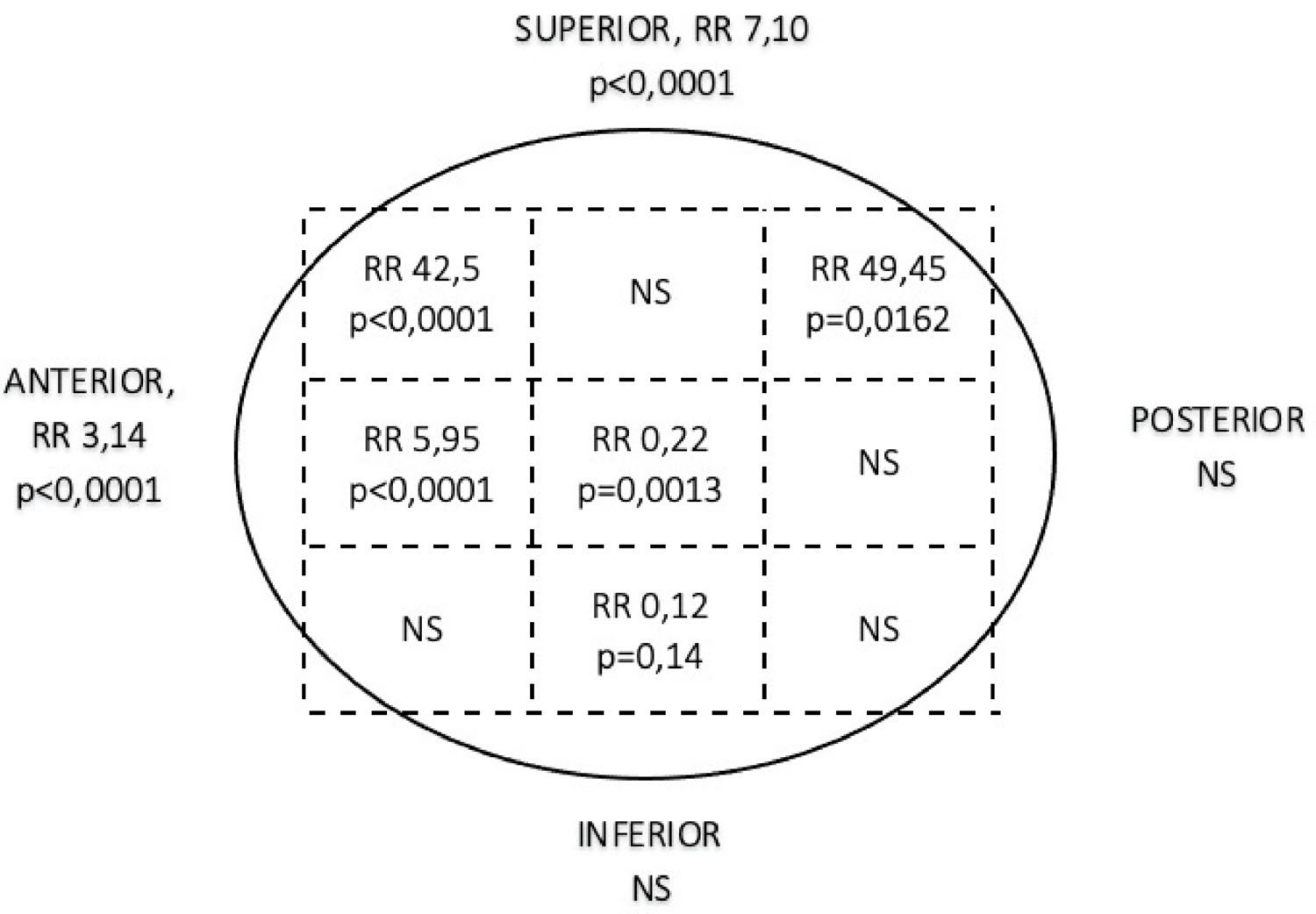
Distribution of relative risks according to the position of the cervicocapital screw

3.1. Number of errors The mean number of errors was 3.00 (1–5; 1.17) in group B and 0.961 (0–6; 1.07) in group AB. This difference was significant (*p* < 0.001).
3.2. Type of errors:
  3.2.1. Entry point: At least one entry point error was present in 19 cases in group B (63%) and in 130 cases in group AB (25%) (*p* < 0.0001). The presence of a single entry point error significantly increased the risk of cut-out occurrence (RR = 2.48; *p* < 0.0001). A too-lateral entry point was observed in 6 cases in group B (20%) versus 15 cases in group AB (2.9%) (*p* < 0.0001). A too-posterior entry point was observed in 9 cases in group B (30%) versus 76 cases in group AB (15%) (*p* = 0.02). The relative risks for cut-out were increased by 6.8 for a too-lateral entry point (*p* < 0.0001) and by 2.01 for a too-posterior entry point (*p* = 0.0190). A too-medial entry point was observed in 19 cases in group AB (3.7%) and in 1 case in group B (3.3%). A too-anterior entry point was observed in 4 cases in group AB (0.74%) and none in group B (0%). A too-medial or too-anterior entry point was not significantly associated with cut-out (*p* = 0.9 and *p* = 1, respectively).
  3.2.2. Cervicocapital screw: All patients (100%) in group B had at least one error versus 292 (57%) in group AB (*p* < 0.001). The presence of at least one CCS error significantly increased the risk of cut-out occurrence (RR = 1.74, *p* < 0.0001). The CCS was centered in both anteroposterior and lateral views in 304 (60%) cases in group AB and in 4 (13%) cases in group B (*p* < 0.0001). A centered CCS reduced the risk of cut-out occurrence, acting as a protective factor (RR = 0.22, *p* = 0.0013). The CCS was located in the anterior third of the femoral head in 17 cases (57%) in group B and in 26 (5.1%) cases in group AB (*p* < 0.0001), and in the superior third in 12 (40%) cases in group B and in 29 (5.1%) cases in group AB (*p* < 0.0001). The relative risks for cut-out occurrence were increased by 3.14 and 7.10, respectively (*p* < 0.0001).
  3.2.3. Tip-apex distance: The mean TAD was 24.87 mm (6–45; 8.09) in group B and 19.92 mm (0–50; 5.90) in group AB (*p* < 0.0001). The TAD was greater than 25 mm in 14 cases in group B (47%) and in 86 cases in group AB (17%) (*p* < 0.001). A TAD > 25 mm increased the risk of cut-out occurrence compared to a TAD < 25 mm (RR = 2.76, *p* < 0.0001).
  3.2.4. Cartilage-CCS distance: The cartilage-screw distance was less than 5 mm in 7 (23%) cases in group B and in 36 (7.1%) cases in group AB (*p* = 0.0018). A cartilage-screw distance < 5 mm increased the risk of cut-out occurrence (RR = 3.30, *p* = 0.0012).
  3.2.5. Distal screw: A distal screw error was found in 16 osteosyntheses in group AB (3.1%) but in none in group B. No significant results were found regarding distal screw errors (*p* = 0.33). The relative risk distribution of cut-out occurrence according to CCS positioning in Cleveland zones is detailed in Fig. 4.

**Fig. 5:**
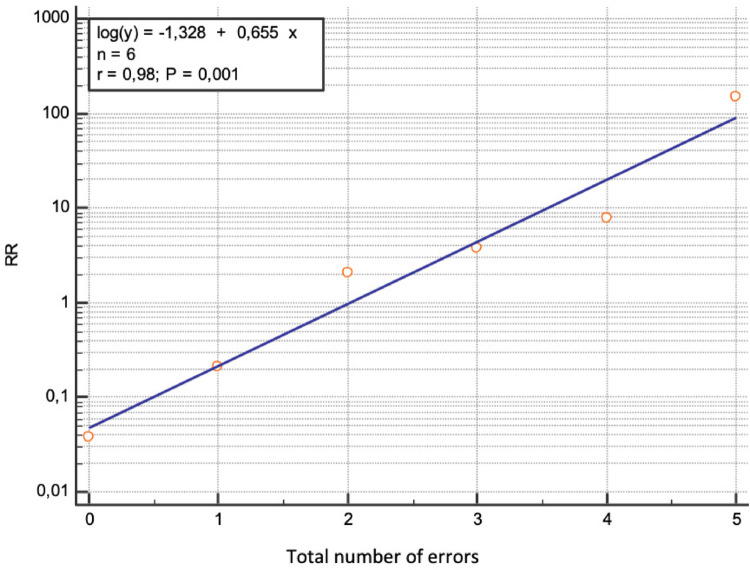
Analysis of the relative risk of sweep based on the total number of errors

3.2.6. Accumulation of errors: When technical errors accumulated in an intramedullary nail, the relative risk (RR) of cut-out occurrence increased exponentially (*p* < 0.0001), from 0.21 for one error to 148.35 (*p* < 0.0007) for five errors (Fig. 5). This significant exponential increase was also observed with accumulated entry point errors (0 errors: RR = 0.49, *p* < 0.003; 1 error: RR = 1.71, *p* = 0.03; 2 errors: RR = 6.47, *p* < 0.0001) and with accumulated CCS errors (0 errors: RR = 0.03, *p* < 0.01; 2 errors: RR = 3.77, *p* < 0.0001; 3 errors: RR = 15.11, *p* < 0.0001; 4 errors: RR = 49.45, *p* < 0.01).

3. Multivariate analysis (Table 2)

**Table 2:** Multivariate Analysis – Independent Predictive Factors for Sweep

Independent Variables	*p*
Intrafocal entry point	0,0310
Cervicocephalic screw localized in Zone 1 (anterosuperior)	< 0,0001
Cervicocephalic screw localized in Zones 1-4-7 (anterior)	< 0,0001
TAD > 25 mm	0,0003

TAD: tip apex distance

Four variables were significantly and independently associated with an increased risk of cut-out occurrence: CCS positioning in the anterior third of the femoral head (zone 1-4-7) (*p* < 0.0001), CCS positioning in zone 1 of the femoral head (*p* < 0.0001), TAD > 25 mm (*p* = 0.0003), and an intra-focal entry point (*p* = 0.03).

## Discussion

1- Cut-out rate

The CCS cut-out is one of the most common complications following SIN. It usually requires surgical revision with total hip arthroplasty, which carries a risk of complications in frail patients [16]. The 5.6% cut-out rate in this study is consistent with the literature. Morvan et al. reported a CCS cut-out rate of 5.7% (*n* = 13/228) [13]. Bojan et al. reported a CCS cut-out rate of 2.3% in a total of 3,066 SIN procedures [5].

In the series of 47 patients by Fernandez et al., the addition of cementation to the cervicocapital screw prevented the occurrence of cut-out [17].

However, unlike most published series reporting mid- to long-term outcomes, our analysis was limited to early mechanical failures within the first 3 months, which may partly explain the observed cut-out rate. Although systematic follow-up beyond 3 months was not available for all patients, three additional late cut-outs were identified between 2019 and 2021(at a median of 4 months after surgery) during non-systematic clinical or radiological follow-up, outside the framework of the present study. Consequently, the true incidence of late mechanical failure is likely underestimated, particularly in the context of substantial loss to follow-up.

2- Accumulation of technical errors

When technical errors in an SIN accumulate, the relative risk (RR) of CCS cut-out increases exponentially and significantly (*p* < 0.0001), rising from 0.21 for one error to 148.35 (*p* = 0.0007) for five errors (Fig. 5).

Three major risk groups for cut-out emerge based on the accumulation of technical errors:

- A “low-risk” group, corresponding to 0 or 1 error, where there is no significant increase in cut-out risk.
- An “intermediate-risk” group, with 2 or 3 accumulated errors, where the RR is 2.07 (*p* = 0.008) and 3.77 (*p* = 0.0001), respectively.
- A “high-risk” group, with 4 or 5 accumulated errors, where the RR is 7.84 (*p* < 0.0001) and 148.35 (*p* = 0.0007), respectively.

To our knowledge, no previous study has specifically explored this cumulative approach.

3- Cervicocapital screw position

The positioning of the CCS remains a subject of debate in the literature. There is no consensus on whether an anterior or posterior placement is more detrimental [5, 18].

Historically, Parker advised against placing the CCS in the posterosuperior quadrant (Cleveland zone 3) due to the higher rate of cut-out associated with this position [18]. Conversely, Baumgaertner discouraged positioning the CCS in the anterosuperior and inferoposterior quadrants (Cleveland zones 1 and 9) [19].

Similarly to Pervez [20], we advise against positioning the CCS in the anterior third (Cleveland zones 1-4-7) of the femoral head, as this placement significantly increased the risk of cut-out in our series (RR = 3.14; *p* < 0.0001), particularly in the anterosuperior quadrant (Cleveland zone 1) (RR = 42.5; *p* < 0.0001). However, our data did not show a significant effect of an overall posterior third placement (Cleveland zones 3-6-9) on the risk of cut-out (*p* = 0.21). Nevertheless, an isolated RR of 49.45 (*p* = 0.0162) was observed in the posterosuperior quadrant (Cleveland zone 3), though this result should be interpreted with caution given that only one case in our series was located in zone 3.

A centered orientation in the lateral view significantly reduces the risk of displacement and mitigates femoral head rotation around the screw [21–23]. However, its role in reducing the risk of cut-out remains debated when the CCS is positioned inferiorly [5]. We confirm the importance of a centered screw placement due to its protective effect (RR = 0.22; *p* = 0.0006) and acknowledge a weaker protective effect for an inferior placement, with a relative risk of 0.12 (*p* = 0.14).

Our study identified three Cleveland zones where CCS placement significantly increased the risk of cut-out:

- Zone 1: Anterosuperior (RR = 42.5; *p* < 0.0001).
- Zone 3: Posterosuperior (RR = 49.45; *p* < 0.0001).
- Zone 4: Pure anterior (RR = 5.95; *p* < 0.0001).

The placement of the CCS in zones 2, 6, 7, and 9 does not seem to significantly influence the risk of cut-out. Conversely, placement in zones 5 and 8 appears to reduce the risk (RR = 0.22; *p* = 0.0013 and RR = 0.12; *p* = 0.14, respectively). These findings align with the literature, which recommends a “center-center” or “center-inferior” CCS position [21–23].

4- Tip-apex-distance

The results of this study confirm the influence of a TAD > 25 mm on the risk of CCS cut-out [13, 19, 24, 25]. The mean TAD was 24.9 mm (range: 6–45 mm; SD: 8.09) in the B group and 19.9 mm (range: 0–50 mm; SD: 5.9) in the AB group, with this difference being significant (*p* < 0.0001). Moreover, the risk of CCS cut-out increased (RR = 2.76; *p* < 0.0001) when the TAD was > 25 mm.

Additionally, we observed that when the cartilage-to-screw head distance was < 5 mm, the risk of CCS cut-out also increased (RR = 3.30; *p* = 0.0012).

5- Type of fracture

The fracture type is recognized as a factor influencing the risk of secondary displacement and CCS cut-out [26]. Indeed, unstable fractures (AO/OTA 31A2 and 31A3) are at the highest risk of cut-out [5]. Furthermore, Haidukiewych [26] reported in 2001 a CCS cut-out rate of 12.7% in patients with oblique type 31A3 fractures, which are considered unstable.

However, in our series, we did not find any significant influence of fracture type on the occurrence of CCS cut-out, nor did we observe a difference in angulation between the “B” and “AB” groups.

6- Reduction quality

Numerous studies have highlighted the relationship between reduction quality and CCS cut-out [13, 20, 27]. Our findings align with these studies, showing that the risk of CCS cut-out decreases with a “good” reduction (RR = 0.36; *p* = 0.0003), whereas it increases with a “satisfactory” reduction (RR = 3.86; *p* < 0.0001). However, a “poor” reduction did not significantly increase the risk in our series (*p* = 0.23). This result seems contradictory and is likely due to a small sample size in this subgroup (*n* = 5; 0.1% of the AB group and *n* = 1; 3.3% of the B group).

7- Limits

The main limitation of this study is its retrospective design.

Radiographic signs of osteoporosis were not analyzed using the Singh Index, which could be considered a confounding bias. However, some authors report a weak correlation between osteoporosis and this index [28, 29].

Additionally, four different types of intramedullary nails were used, potentially introducing selection bias. However, the principles and techniques were largely similar, and no significant differences in outcomes were observed [30].

The two study groups were not comparable in terms of patient age. The mean age was higher in the cut-out group, which could introduce a confounding bias. However, a multivariate analysis was performed to address this issue, and age was not identified as an independent risk factor for CCS cut-out.

The short follow-up limits the analysis to early mechanical failures and may underestimate late cut-out rates.

Surgical techniques and implants have evolved since the study period, which may limit extrapolation to current practice.

Baseline characteristics of excluded patients could not be fully analyzed, which may introduce attrition bias, particularly in this frail elderly population.

- Highlights.

This study is the only one to comprehensively evaluate all technical errors in SIN fixation across a large cohort (*n* = 540). Previous studies have primarily focused on CCS positioning and TAD without detailing other potential sources of error (e.g., entry point, distal screw placement) [5, 13, 19, 24].

Additionally, this study highlights the cumulative impact of technical errors on mechanical failure. Specifically:

- An intra-focal nail entry point, an excessively anterior CCS placement, and a TAD > 25 mm increase the risk of secondary displacement.
- The accumulation of technical errors exponentially increases the risk of CCS cut-out.

These risk factors were analyzed in the context of uncemented cephalomedullary fixation. The impact of technical errors in augmented nailing remains an open question.

## Conclusion 

In this retrospective study, the early cervicocapital screw cut-out rate following cephalomedullary nailing was 5.6%, which is consistent with previously published data. Several technical factors were found to be associated with an increased risk of early mechanical failure, including an intra-focal nail entry point, an anterior cervicocapital screw position, and a tip–apex distance greater than 25 mm.

In addition, our results suggest that the accumulation of technical errors is associated with a marked increase in the risk of early cut-out. This cumulative approach should be interpreted as a conceptual model reflecting the progressive loss of mechanical safety margins, rather than as a biomechanically weighted predictive score.

These findings must be interpreted in light of several limitations, including the retrospective design, the short follow-up limited to early mechanical complications, the study period (2012–2018), and the absence of data on bone quality. Furthermore, loss to follow-up may have led to an underestimation of late mechanical failures.

Within these limitations, this study highlights the importance of meticulous surgical technique in cephalomedullary fixation and suggests that minimizing the accumulation of technical errors may reduce the risk of early mechanical failure. Further prospective studies with longer follow-up are required to confirm these associations and to assess their relevance in contemporary practice.

## Data Availability

No datasets were generated or analysed during the current study.
